# A four-generation family transmission chain of COVID-19 along the China–Myanmar border in October to November 2021

**DOI:** 10.3389/fpubh.2022.1004817

**Published:** 2022-11-17

**Authors:** Xiangyu Yan, Wei Xiao, Saipeng Zhou, Xuechun Wang, ZeKun Wang, Mingchen Zhao, Tao Li, Zhongwei Jia, Bo Zhang, Tiejun Shui

**Affiliations:** ^1^School of Public Health, Peking University, Beijing, China; ^2^Mang City Center for Disease Control and Prevention, Mang, China; ^3^Chinese Center for Disease Control and Prevention, Beijing, China; ^4^Center for Intelligent Public Health, Institute for Artificial Intelligence, Peking University, Beijing, China; ^5^Center for Drug Abuse Control and Prevention, National Institute of Health Data Science, Peking University, Beijing, China; ^6^Peking University Clinical Research Institute, Beijing, China; ^7^Yunnan Center for Disease Control and Prevention, Kunming, China

**Keywords:** COVID-19, transmission chain, China-Myanmar border, refugees, outbreak

## Abstract

**Background:**

Foreign imported patients and within-household transmission have been the focus and difficulty of coronavirus disease 2019 (COVID-19) prevention and control, which has also posed challenges to border areas' management. However, household transmission caused by foreign imported cases has not been reported in China's border areas. This study aimed to reveal a clear family clustering transmission chain of COVID-19 caused by contact with Myanmar refugees along the China–Myanmar border during an outbreak in October to November 2021.

**Methods:**

During the outbreak, detailed epidemiological investigations were conducted on confirmed patients with COVID-19 and their close contacts in daily activities. Patients were immediately transported to a designated hospital for treatment and quarantine, and their close contacts were quarantined at designated sites. Regular nucleic acid testing and SARS-CoV-2 antibody testing were provided to them.

**Results:**

A clear four-generation family clustering transmission involving five patients with COVID-19 was found along the China–Myanmar border. The index case (Patient A) was infected by brief conversations with Myanmar refugees across border fences during work. His wife (Patient B) and 9-month-old daughter (Patient C) were second-generation cases infected by daily contact with him. His 2-year-old daughter (Patient D) was the third-generation case infected by her mother and sister during quarantine in the same room and then transmitted the virus to her grandmother (Patient E, the fourth-generation case) who looked after her after Patients B and C were diagnosed and transported to the hospital. The household secondary attack rate was 80.0%, the average latent period was 4 days, and the generation time was 3 days. Ten of 942 close contacts (1.1%) of this family had positive IgM antibody during the medical observation period. In total 73.9% (696/942) of them were positive for IgG antibody and 8.3% (58/696) had IgG levels over 20 S/CO (optical density of the sample/cut-off value of the reagent).

**Conclusion:**

This typical transmission chain indicated that it is essential to strengthen COVID-19 prevention and control in border areas, and explore more effective children care approaches in quarantine sites.

## Introduction

The highly infectious SARS-CoV-2 Delta variant strain (B.1.617.2) was first found in India and spread rapidly around the world and was listed as one of the variants of concern (VOC) by the World Health Organization (WHO) (1, 2). Since the first local coronavirus disease 2019 (COVID-19) patient infected with SARS-CoV-2 Delta VOC was detected in Guangzhou, China on May 21, 2021, Delta VOC has spread to more than 50 cities across China and led to 11 outbreaks, all of which were caused by imported patients with COVID-19 (3). A case in point was the outbreak in Nanjing, China, which was caused by passengers on an international flight transmitting to a cabin cleaner at Nanjing Lukou Airport (3, 4). Household transmission triggered by imported cases was the main pattern of local outbreaks. Close contact in households made it the ideal route for virus transmission. Previous studies reported a relatively high household secondary attack rate in China during the pandemic of COVID-19, reaching 15.6% and 12.4–17.1% in Wuhan and Guangzhou, respectively (5, 6). However, the household transmission of COVID-19 in China caused by foreign imported patients has not been systematically reported.

Mang City is a city in Yunnan Province, China, bordering Myanmar. It is near the Kokang region of northern Shan State in Myanmar, where civil wars often occur, resulting in poor accessibility to basic health services and frequent influx of war refugees into the China–Myanmar border area (7, 8). The China–Myanmar border area is also one of the areas with the greatest risk of overland importation of infectious diseases to China; for instance, the incidence of malaria was highest in the city along the China–Myanmar border (9–11). Similarly, the risk of imported COVID-19 cases from the border also threatened China (12). This study aimed to reveal a clear family clustering transmission chain of SARS-CoV-2 Delta VOC caused by contact with Myanmar refugees along the China–Myanmar border during an outbreak in October 2021, which could provide practical implications for COVID-19 prevention and control in border areas.

## Methods

### Study procedure

On 17 October 2021, a confirmed patient with COVID-19 was reported by a local fever clinic in Mang City, Yunnan Province, China. On the same day, the confirmed COVID-19 case was immediately transported to a designated hospital for treatment and quarantine. An epidemiological investigation was conducted to obtain detailed information on the source of infection, disease onset process, and close contacts. Subsequently, his family members and other close contacts were also quarantined in designated hotels or at home for at least 14 days for medical observation. All confirmed patients and close contacts received epidemiological investigation by the local Center for Disease Control and Prevention (CDC) on the day they were found. Nasopharyngeal swabs nucleic acid testing (NAT) and serum SARS-CoV-2 antibody testing (IgM and IgG) were performed regularly during their medical observation period. Local CDC staff also collected samples and conducted NATs from patients' working and living environments. Two types of close contacts were included in management. One was the primary close contacts who had direct contacts or ever stayed in a confined space with patients with COVID-19, including family members, work colleagues, and people who attended the same clinic (13). The other was the secondary close contacts who were close contacts of the primary close contacts of patients with COVID-19. Once they were confirmed as COVID-19 infections by NAT, they were transported from the quarantine hotel or home to the designated hospital. Patients' and close contacts' COVID-19 vaccination records were extracted by matching ID numbers from the vaccination database.

For the laboratory testing, real-time reverse transcriptase–polymerase chain reaction (RT-PCR) targeting at ORF1ab and N genes was conducted using the Novel Coronavirus 2019 Nucleic Acid Test Kit (Bojie Medical Technology, Shanghai Municipality; Daan Gene Company, Guangzhou; Wuhan Easy. Diagnosis Biomedicine Company, Wuhan, China), of which the test result values have little difference among the three test kits. The average result values of the three test kits were reported in this study. The detection limit of the cycle threshold (Ct) of RT-PCR was 40, a Ct value of less than 40 was considered NAT positive. The lower Ct value reflected a higher viral load and higher transmissibility. For NATs of patients, the results were reported with exact Ct values because of the need for continuous clinical observation; for NATs of environmental samples, only positive or not of results were reported. SARS-CoV-2 antibodies were tested using the Anti-SARS-CoV-2 Rapid Test Kit (Antubio Diagnostics Company, Zhengzhou, China). A value of over 1 S/CO (optical density of the sample/cut-off value of the reagent) was positive for IgM and IgG tests. The biological samples of confirmed patients were also sent to China CDC for further virus genotyping. We received the laboratory test results from China CDC's report for transmission chain inference. Compared with the reference genome sequence of Wuhan (GenBank No.NC_045512), the China CDC's report showed the subtype of the SARS-CoV-2, mutation site, and homology between patients. Considering that COVID-19 outbreaks along the China–Myanmar border in China were mainly caused by imported cases from Myanmar (14), the gene sequences of the Chinese patients were also compared with those collected in Myanmar.

The ethical approval was provided by the Mang City Center for Disease Control and Prevention, Yunnan, China.

### Statistical analysis

The latent period of each patient was calculated as the duration from the earliest possible date of exposure to the date of first symptoms onset (for patients with symptomatic) or NAT positive (for patients with asymptomatic). The generation time was defined as the time interval between the date of the previous generation infection (symptoms onset or NAT positive) and the date of the next-generation infection. The average latent period and generation time were also calculated to estimate the whole family clustering transmission situation. In addition, based on the antibody testing results of close contacts, the positive rate of IgM and IgG antibodies of close contacts were calculated, respectively.

## Results

### Basic information about this family

**Figure 1:**
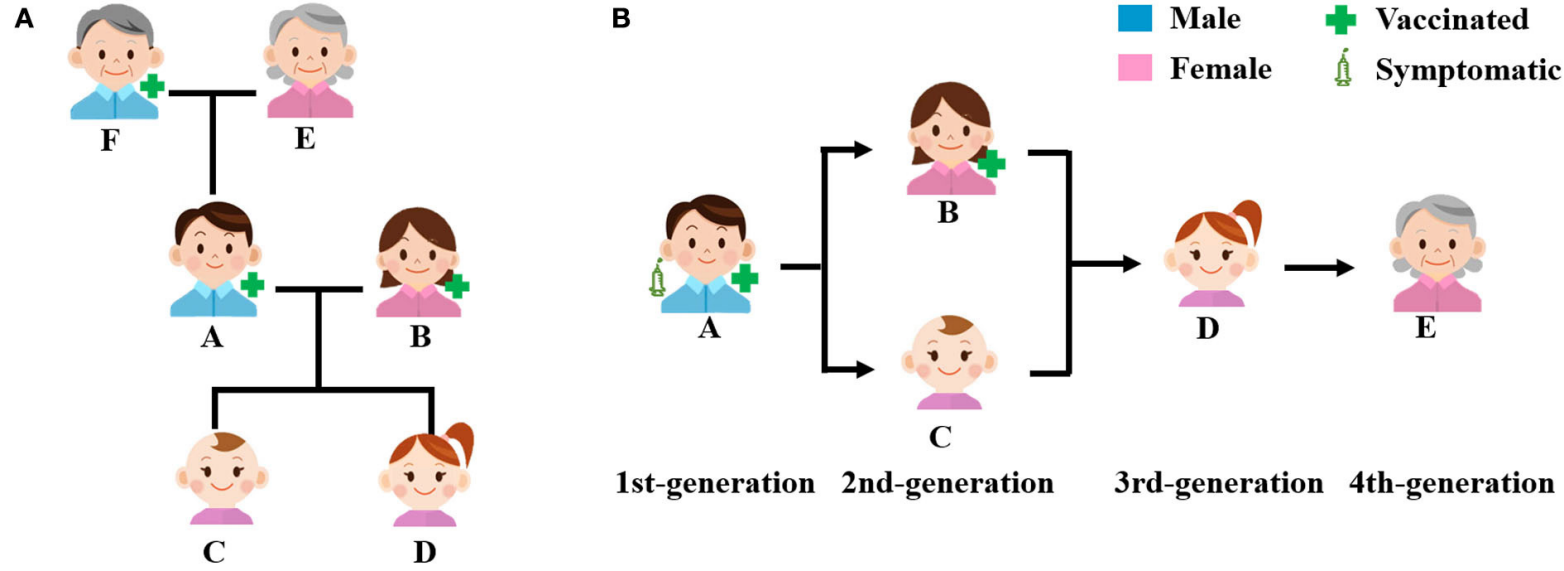
Index case's family members and the transmission chain. **(A)** Relationships of index case's family members and **(B)** transmission chain.

The index case (Patient A) of the family clustering transmission was a 27-year-old Chinese male worker on the Chinese side of the China–Myanmar border in Mang City. He lived in the community near his workplace with his wife (a 27-year-old Myanmar female living in China [B]), two daughters (a 9-month-old baby [C], and a 2-year-old girl [D]), mother (a 54-year-old Chinese female [E]), and father (a 53-year-old Chinese male [F]) (Figure 1A, Table 1). His wife was a housewife, and his parents ran a wine-making business at home. Moreover, his father was the village headman, who was often in charge of the village affairs. Patient A, his wife, and his father have received two doses of inactivated COVID-19 vaccine. His two daughters were not vaccinated because they did not meet the age requirements for vaccination. Additionally, his mother was not vaccinated because of contraindications to vaccination (Table 1).

**Table 1:** Characteristics of the index case and his family members.

					**First positive nucleic acid test**			**Fully COVID−19 vaccination**	**Antibody testing results**			
						**Ct value**			**First time**		**Last time**	
**Family member**	**Sex**	Age^*^	**Nationality**	**COVID−19 patient**	**Date**	**ORF1ab gene**	**N gene**		**Date**	**Results (S/CO)**	**Date**	**Results (S/CO)**
A	Male	27 y	China	Yes	Oct 17, 2021	16.44	14.32	Yes	Oct 19, 2021	IgM: 3.59 (+); IgG: 0.13 (–)	Oct 28, 2021	IgM: 11.28 (+); IgG: 6.29 (+)
B	Female	27 y	Myanmar	Yes	Oct 18, 2021	30.86	30.26	Yes	Oct 19, 2021	IgM: 3.01 (+); IgG: 0.57 (–)	Oct 28, 2021	IgM: 2.59 (+); IgG: 7.26 (+)
C	Female	9 m	China	Yes	Oct 18, 2021	20.18	21.48	No	Oct 19, 2021	IgM: 0.01 (–); IgG: 0.07 (–)	Oct 31, 2021	IgM: 1.98 (+); IgG: 1.56 (+)
D	Female	2 y	China	Yes	Oct 24, 2021	14.99	13.86	No	Oct 21, 2021	IgM: 0.01 (–); IgG: 0.04 (–)	Nov 5, 2021	IgM: 0.91 (–); IgG: 2.23 (+)
E	Female	54 y	China	Yes	Oct 24, 2021	36.87	35.18	No	Oct 21, 2021	IgM: 0.03 (–); IgG: 0.04 (–)	—	—
F	Male	53 y	China	No	—	—	—	Yes	Oct 21, 2021	IgM: 0.11 (–); IgG: 12.43 (+)	—	—

* y, years; m, months.

Since Patient A was diagnosed on 17 October 2021, as of 6 November 2021, four family contacts of Patient A were diagnosed as asymptomatic patients with COVID-19 successively. All five patients were infected with the same SARS-CoV-2 Delta VOC with 44 mutation sites based on virus genotyping and gene sequencing. The household secondary attack rate was 80% (4/5). Based on epidemiological investigation and laboratory testing results, we could infer that virus spread in this family for four generations with an average latent period of 4 days and a generation time of 3 days (Figure 1B, Table 1).

### Transmission chain

#### Stage 1. Transmission from Myanmar refugees to patient A

**Figure 2:**
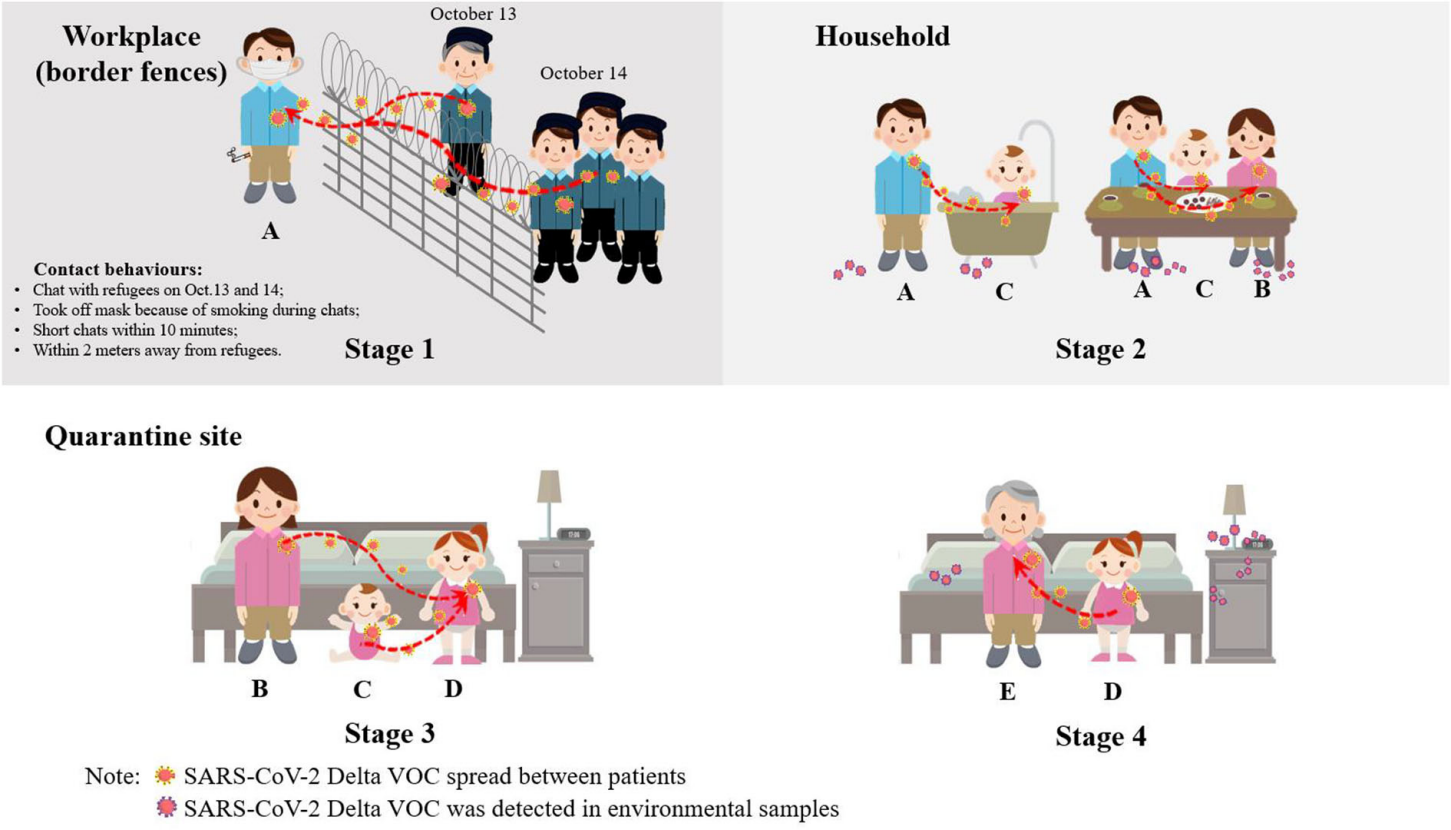
Virus transmission process.

Before Patient A was diagnosed, he self-reported that some flu-like symptoms occurred on 15 October 2021. On 17 October, he was diagnosed as a confirmed patient by regular NAT (Ct value: 16.44 of ORF1ab gene and 14.32 of N gene) (Table 1). According to the epidemiological investigation, he spent most of his time working, living, and eating at a workplace near the border fences and returned home to care for children during breaks. He declared that he had no contact with other patients with COVID-19 or anyone with suspected symptoms before he was diagnosed. Because the community he worked and lived in was close to the China–Myanmar border, residents received regular NATs every week provided by the local CDC for early detection of COVID-19 cases. His last NAT was provided on 14 October 2021, with negative results, which indicated that he was a newly infected case within 1 week. However, according to further review of his workplace video 1 week before his symptom onset, investigators found that Patient A had two brief conversations (within 10 min) with Myanmar refugees across the border fences during work on October 13 (with an old man) and 14 (with several adults approaching in succession), respectively, when Myanmar refugees poured into the border area of Myanmar near the border fences due to the war in northern Myanmar. During the conversations, Myanmar refugees did not wear facemasks and stood closely with Patient A within 2 m, and Patient A sometimes removed the mask because of smoking (Figure 2). The transmission was confirmed by gene sequencing. During the epidemiological investigation and disinfection of Patient A's workplace, some Myanmar refugees sought health services from China's local CDC's staff across the border fences and were taken nasopharyngeal swabs for NAT and further gene sequencing. The same SARS-CoV-2 Delta VOC gene sequence with 44 mutation sites was found among Patient A and the Myanmar refugees' COVID-19 cases.

#### Stage 2. Transmission from patient A to patients B and C

After Patient A was diagnosed and transported to the designated hospital, his family was also transported to the designated quarantine hotel for medical observation on 17 October. On 18 October, Patient A's wife (Patient B) and second daughter (Patient C) were diagnosed as newly infected asymptomatic cases with positive NAT results because their last NAT results on October 14 were negative. Their antibody testing results could also prove it. Patient B had only a positive IgM result one day after being diagnosed, but both IgM and IgG presented positive 10 days later. Patient C had negative IgG and IgM antibody results on 19 October, but both had positive results on 31 October 2021 (Table 1). Patient B had no contact with other patients with COVID-19 except her husband. Patient C's daily contacts were mainly with her family. Therefore, it can be inferred that these two patients were the secondary cases in the family. Between 12 October and 15 October, Patient A did not live at home, but he went home to help her wife bathe the baby during mealtime every day, and he went to the bank with his wife and child on 14 October. On 16 October, Patients A, B, and C ate and slept together overnight, and two NAT-positive environmental samples were detected on the bedroom floor and the head of the bed. These direct contacts made the spread of the virus possible (Figure 2).

#### Stage 3. Transmission from patients B and C to patient D

When the family members were transported to the designated quarantine hotel, Patient A's parents were quarantined in separate rooms. Since the two daughters of Patient A were too young to take care of themselves, they were quarantined in the same room of the quarantine hotel with their mother (Patient B) until 19 October, when Patients B and C were diagnosed as confirmed cases and were transported to the designated hospital. After that, the elder daughter was still quarantined in the hotel and was provided regular NATs every 3 days. Her NATs results were negative on 18 October and 21 October. However, the NAT results turned positive on 24 October without symptoms occurring. The girl became the fourth patient with COVID-19 (Patient D). The negative NAT result on 21 October showed that she could have been in the latent infection period. Except that the date of Patient D's NAT positive was about 1 week later than Patients B and C, the epidemiological investigation also showed Patient D was less likely to be transmitted by Patient A because Patient D was usually taken care of by her grandparents and the direct contacts between Patients A and D were rare on these days. Therefore, Patient D was considered as the third-generation case transmitted by her mother and younger sister (the second-generation cases Patients B and C) during the same-room quarantine (Figure 2).

#### Stage 4. Transmission from patient D to patient E

On 24 October, the same day that Patient D was diagnosed, Patient A's mother (Patient E) was also diagnosed with an asymptomatic case. Patient E's last negative NAT result was on 21 October, suggesting that she was also newly infected. Similar to Patient D, Patient E was less likely to be transmitted by Patient A for two reasons. First, the date of Patient E's NAT positive was about 1 week later than secondary cases B and C; second, the direct contact between Patients A and E were rare because Patients A and E did not live in the same room at home, and Patient E spent her time running the wine-making business, but Patient A spent very little time at home after October 12 and did not help Patient E with her work. In addition, the possibility of the transmission from Patient B or C to Patient E was also small because after the families were transported to quarantine, Patient E was not quarantined in the same room as Patients B and C, and they had no direct contact during the quarantine. The most possible transmission was from Patient D to E because when Patients B and C were diagnosed and were transported from the hotel to the designated hospital, Patient D was sent to her grandmother's (Patient E) room since she was too young to stay alone. At that time, she had already been infected but was in the latent period. Two pieces of evidence could prove the transmission. First, environmental sample testing on October 25 showed that in the quarantine room of Patients D and E, the samples collected from the Patient D's slippers, toilet, floor, table, trash can, bedding, and cup showed positive NAT results, which showed the possibility of transmission in this room. Second, changes in the Ct values of Patients D and E also provided clues of transmission. Considering the process of virus replication and elimination in the human body, the viral load was low at the early stages of infection, and then decreased after an increasing period (15). Therefore, the Ct values presented a U-shaped curve. For Patient E, we could clearly observe the U-shaped Ct value curve after October 24; however, the Ct value of Patient D kept going up, suggesting that Patient D got infected earlier than Patient E (Supplement Figure S1). Therefore, Patient E could be considered as the fourth-generation case transmitted by her elder granddaughter (the third-generation Patient D) during the quarantine in the same room (Figure 2).

### Medical observation of patients' close contacts

A total of 946 close contacts of these patients were found through epidemiological investigation and travel history big data, including 134 primary close contacts and 812 secondary close contacts. Except for four families of Patient A were diagnosed as patients with COVID-19 later, no other patients with COVID-19 were confirmed by NAT among these 942 close contacts. However, 10 of the 942 close contacts (1.1%) had positive IgM antibodies during their medical observation period. In total 73.9% (696/942) of them had positive IgG antibody and 8.3% (58/696) of the IgG-positive persons had an IgG level over 20 S/CO.

It was worth noting that as high-risk primary close contacts, Patient A's father and his colleagues were not diagnosed as patients with COVID-19, which might partly owe to all of them having been vaccinated against COVID-19. Another reason was the characteristics of their direct contacts. After 12 October, Patient A started to work on duty, while his father had to handle village affairs and the wine business, and the direct contact time between the two was very short. For contact with his colleagues, Patient A wore facemasks during work hours and did not live in the same room as his colleagues.

## Discussion

This is the first study to reveal a clear transmission chain of SARS-CoV-2 Delta VOC along the China–Myanmar border, which mixed the cross-border overland transmission and family clustering transmission. Because of the large cross-border population movement in border areas, tracing the source of SARS-CoV-2 transmission is always difficult. This study is the first to tease out the chain of transmission at its source along the China–Myanmar border, which could contribute to COVID-19 prevention and control in border areas.

The index case was infected by Myanmar refugees who poured into the border area of Myanmar near the border fences due to the war in northern Myanmar. The infection process was only through brief conversations across the border fences, indicating that the brief companion of time and space outdoors was enough for SARS-CoV-2 transmission. Previous studies have indicated that SARS-CoV-2 could spread through aerosols, survive, and maintain infectivity for up to 16 h (16, 17). Coughing and sneezing could help SARS-CoV-2 spread through the small droplets for a potential long distance of up to 7–8 m (17, 18).

Problems caused by a refugee in border areas are major challenges for many countries. Recently, Poland–Belarus border refugee crisis has intensified, and many refugees from the Middle East have poured into the border areas of Poland and Belarus, trying to reach Western European countries (19). The gathering of crowds increased the risk of COVID-19 spreading among refugees, and also added to the pressure on border epidemic prevention and control in countries of entry. The hometowns of the refugees were war-torn countries that often did not have the financial capacity to provide enough COVID-19 prevention and testing services. Therefore, international exchange as well as humanitarian aid, coordination, and strategy should be strengthened, which requires to collaborate between governments, the WHO, the International Red Cross, various foundations, and other departments to provide not only basic living necessities but also COVID-19 vaccinations, testing, and treatment services (20). For border areas of entry countries, regular disinfection around the border fences needs to be implemented. It is also essential to strengthen publicity and education, including wearing masks and other protective equipment during work and outdoors, being proactive with COVID-19 vaccination, and reducing close proximity to border fences and contacts with refugees. In addition, providing regular NAT to workers and residents in border areas can effectively detect COVID-19 cases in time to prevent the further spread of the epidemic.

Quarantine site management is also a global issue, with several quarantine system failures reported in Australia and New Zealand (21). In this transmission chain, Patient D, the 2-year-old girl, was the pivot. After being infected by her mother and sister, she transmitted the virus to her grandmother in the quarantine site, reflecting that the children's care would be an important issue in management. Compared with home quarantine, centralized quarantine management is indeed essential for controlling the spread of the virus as soon as possible and helps to carry out the thorough disinfection of COVID-19 patients' homes and workplaces. In this study, quarantine in a separate room in a centralized quarantine site effectively prevented the index case's father from getting infected. However, the children who could not take care of themselves became the unavoidable source of infection in the quarantine site. Therefore, it is imperative to explore more effective mechanisms for the management of children in quarantine sites, including developing standards to determine whether children need to be separated from their families in quarantine, arranging specialized healthcare workers to take care of children in quarantine, and providing psychological counseling and care services for mothers and children.

In addition, though no other close contacts of these patients were diagnosed as COVID-19 cases by NAT, 10 of them had positive IgM test results and 58 had IgG results of over 20 S/CO, which was considered as the standard for identifying probable cases in China–Myanmar border area (12). Previous studies also reported potentially infected cases with negative NAT results but positive antibody results in Guangdong province and Liaoning province, China (22, 23). It is worth exploring further whether they were actually infected and could conceal transmission of the virus.

Our study also had a limitation. Because of the high mobility of refugees and the difficulty in communication, it was impossible to identify the exact person who transmitted SARS-CoV-2 to Patient A. However, the homology of the sequencing indicated that Patient A was infected by Myanmar refugees. In conclusion, this study revealed a clear transmission chain of SARS-CoV-2 Delta VOC mixing the cross-border overland transmission and family clustering transmission along the China–Myanmar border. It is essential to strengthen COVID-19 prevention and control in border areas and explore more effective children-care approaches in quarantine sites.

## Data availability statement

The original contributions presented in the study are included in the article/Supplementary material, further inquiries can be directed to the corresponding author/s.

## Ethics statement

The studies involving human participants were reviewed and approved by the Mang City Center for Disease Control and Prevention, Yunnan, China. Written informed consent to participate in this study was provided by the participants' legal guardian/next of kin. Written informed consent was obtained from the individual(s), and minor(s)' legal guardian/next of kin, for the publication of any potentially identifiable images or data included in this article.

## Author contributions

TS, SZ, and BZ had full access to all of the data in the study and take responsibility for the integrity of the data and the accuracy of the data analysis. XY and TS contributed to the concept and design. XY, MZ, and BZ contributed to the drafting of the manuscript. XY, XW, and ZW contributed to the statistical analysis. ZJ and BZ obtained funding. WX and SZ contributed to administrative, technical, and material support. TS and BZ contributed to the supervision. All authors contributed to the acquisition, analysis, or interpretation of data and also contributed to the critical revision of the manuscript for important intellectual content.

## Funding

This study was supported by the National Natural Science Foundation of China (Grant Nos.: 72104008, 72174004, 91546203, and 91846302) and the National Key Research and Development Program of China (Grant No.: 2021YFC0863400).

## Conflict of interest

The authors declare that the research was conducted in the absence of any commercial or financial relationships that could be construed as a potential conflict of interest.

## Publisher's note

All claims expressed in this article are solely those of the authors and do not necessarily represent those of their affiliated organizations, or those of the publisher, the editors and the reviewers. Any product that may be evaluated in this article, or claim that may be made by its manufacturer, is not guaranteed or endorsed by the publisher.
