# Supplementary figures and images for: A four-generation family transmission chain of COVID-19 along the China–Myanmar border in October to November 2021
Source: Front Public Health. 2022 Nov 17;10:1004817. doi: 10.3389/fpubh.2022.1004817 (PMC9714430; doi:10.3389/fpubh.2022.1004817)

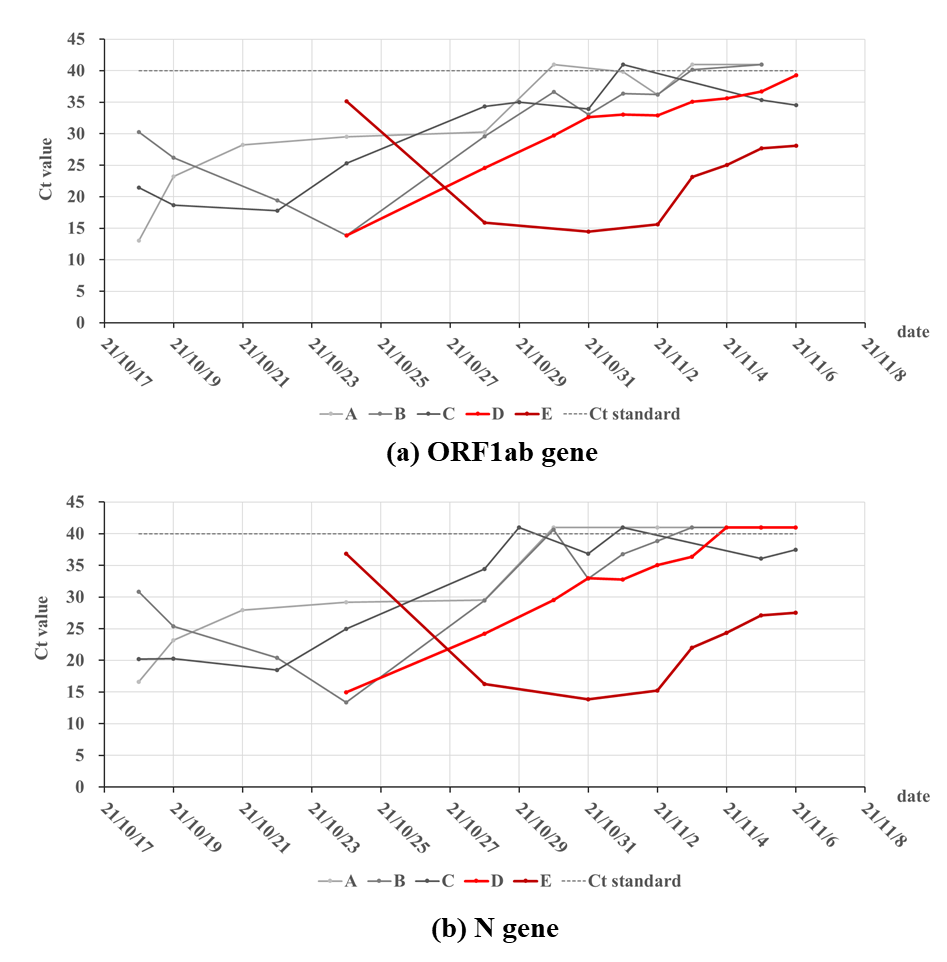


**Supplement Figure S1. Ct value of ORF1ab (a) and N (b) genes of the five COVID-19 patients**

Supplement: Supplementary file 1 [file Data_Sheet_1.DOCX]
